# An integrated metabolomics-flavoromics analysis reveals flavor diversity in indigenous and hybrid cattle via UHPLC-MS/MS and GC×GC-TOF-MS

**DOI:** 10.1016/j.fochx.2025.102801

**Published:** 2025-07-16

**Authors:** Yuanfeng Zhao, Jingrui Zhou, Wenju Luo, Lingling Jiang, Fuzhan Song, Jiang Ran, Haoxiang Xu, Lu Lei, Rong Ai, Bo Yu

**Affiliations:** a Guizhou Institute of Animal Husbandry and Veterinary Sciences, Guizhou Academy of Agricultural Sciences, Guiyang 550005, China; b Key Laboratory of Animal Genetics, Breeding and Reproduction in the Plateau Mountainous Region, Ministry of Education, Guizhou University, Guiyang 550025, China; c Key Laboratory of Animal Genetics, Breeding and Reproduction of Guizhou Province, Guiyang 550025, China; d College of Animal Science, Guizhou University, Guiyang 550025, China; e School of Materials Science and Engineering, Jiangsu University, Zhenjiang 212013, China

**Keywords:** Beef flavor, Volatile organic compounds, Beef breeds, Aroma profile, Sensory profile, Guizhou yellow cattle, GC × GC-TOF MS

## Abstract

This study investigated breed-specific flavor characteristics in Guanling (GL), Sinan (SN), and Simmental-crossbred (XZ) cattle using UHPLC-MS/MS and GC × GC-TOF MS. Specifically, XZ had 73 and 48 differential metabolites compared to GL and SN, respectively, whereas GL and SN showed 31 discriminated metabolites. Notably, these differences were linked to flavor-related metabolic pathways, particularly beta-alanine, histidine, glutathione, and purine metabolism. Regarding the composition of volatile organic compounds (VOCs), GL displayed a higher ketone content (18.58 %), whereas XZ was enriched in alcohol (56.25 %). Key VOCs with the highest flavor contributions across all breeds were 2,3-Butanedione, (E)-2-nonenal, 2-ethyl-1-hexanol, and (E)-2-octenal. However, dimethyl sulfide contributed significantly to GL and SN flavors but not to XZ. Collectively, the 49 differential VOCs led to aroma profiles of breeds varied in “sweet”, “fruity”, “nutty”, and “waxy” characteristics. This comprehensive analysis of non-volatile metabolites and flavor-related VOCs revealed breed-specific profiles and crossbreeding effects, providing insights for improving meat quality.

## Highlights

• UHPLC-MS/MS and GC × GC-TOF MS identified breed-specific metabolites and VOCs.
• Simmental crossbreeding enhanced aroma intensity in Guizhou yellow cattle.
• Guanling cattle showed distinct aroma profiles with a high ketone content.
• Differential metabolites were primarily linked to amino acid metabolism pathways.
• Differential metabolites showed strong correlations with flavor-related VOCs.

## Graphical abstract

**Figure:**
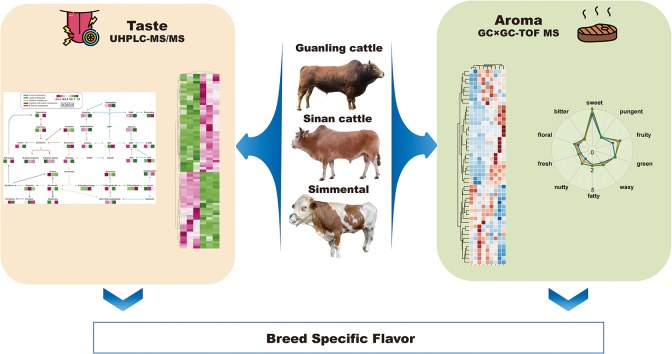

## 1 Introduction

Although the beef flavor is a crucial sensory attribute influencing consumer preference and acceptance, the emphasis on the tenderness of beef has been prominent for decades in many countries, where consumers consider it the primary benchmark for beef palatability. However, increasing evidence suggests that the flavor of beef has emerged as a central contributor to defining consumers' purchase intentions (Kerth et al., 2024). As a complex sensory attribute, beef flavor is a result of sophisticated interactions of water-soluble compounds that generate five basic tastes and volatile organic compounds (VOCs) responsible for aromatic profiles. The beef species-specific flavor is developed during cooking through a combination of various chemical reactions, such as the Maillard reaction and lipid autoxidation (Calkins & Hodgen, 2007; Guth & Grosch, 1993). Recent studies focused on the effect of post-harvest factors such as aging (Liu et al., 2024), cooking (Miller et al., 2023), and packing (Lagerstedt et al., 2011) on beef flavor. However, pre-harvest factors influencing beef constituents and flavor have been less studied. Marbling level, genetics, age, and animal diet have been reported to be critical pre-harvest factors in beef flavor formation (O'Quinn et al., 2024). In addition, animal health, gender, and days on feed can also affect the development of flavor before the animal is harvested. It is believed that the intrinsic non-volatile components in fresh meat, functioning as primary taste modulators and essential flavor precursors, fundamentally determine the taste of cooked meat (Holloway & Wu, 2019). Limited literature has identified the differences in flavor between breeds, and those breed-specific differences were presumably attributed to variations in marbling levels between breeds (O'Quinn et al., 2024). As of 2024, Guanling and Sinan cattle, the two largest Guizhou yellow cattle breeds by population, had a stock of 671,600. Although their meat flavor is the primary reason for consumer preference, the characteristics of the flavor have not been studied.

Additionally, the effect of genetics and breed type on beef flavor traits remains insufficiently understood. Our previous study found variations in the nutritional composition and content of beef among breeds (Guizhou yellow cattle and their Simmental crossbreed), implying unique flavor profiles of different breeds (Zhou et al., 2024). The development of the unique flavor is closely related to the difference in proteins, lipids, and carbohydrates, which contain a myriad of flavor precursors, such as nitrogen-containing compounds (pyrazines, pyrroles), oxygen-containing compounds (alcohols, ketones), and sulfur-containing compounds (thiophenes, sulfides), as well as esters, aldehydes, furans, and hydrocarbons. This evidence supports our hypothesis that the breed type significantly influences beef flavor characteristics, suggesting measurable differences between purebred Guizhou yellow cattle and the Simmental crossbreed. To investigate this hypothesis, omics analyses were employed in this study to achieve a highly sensitive and comprehensive molecular characterization of beef flavor determinants.

Metabolomics enables a comprehensive assessment of the flavor precursors of taste and aroma compounds, which are the end-products integrating intracellular genomic processes and extracellular epigenetic regulation. The flavor-related water-soluble biomarkers can be identified by analyzing the differential metabolites using liquid chromatography-mass spectrometry (LC-MS) based metabolomic profiling (Setyabrata et al., 2021). On the other hand, aromatic VOCs are derived from the complicated biochemical reactions of the endogenous components in meat. While gas chromatography–mass spectrometry (GC–MS) is an established technique for VOC profiling in meat, its effectiveness and accuracy are limited due to the complexity, low content, and instability of VOCs. To improve the detection capability of VOCs, two-dimensional gas chromatography-time-of-flight mass spectrometry (GC × GC-TOF MS) has been developed for the enhancement of resolution and sensitivity (Xiaoyu Wang et al., 2022). Nevertheless, this technique has not been widely applied to study flavor differences in beef, especially beef from different cattle breeds.

Due to a lack of scientific evidence on the specific flavor-related metabolite composition and flavor profile of Guizhou yellow cattle, it is challenging to establish practical grading standards for Guizhou yellow cattle. Furthermore, few studies have explored the effects of breed type on metabolic pathways that may influence beef flavor development. This knowledge gap highlights the need for an investigation into the beef flavor of different cattle breeds. Herein, we applied a flavoromics strategy by combining ultra-high performance liquid chromatography-tandem mass spectrometry (UHPLC-MS/MS) and GC × GC-TOF MS to profile both non-volatile and volatile compounds contributing to beef flavor and reveal the influence of metabolic macromolecules and small odor molecules on the formation of breed-specific beef flavor. Multivariate analysis was employed to analyze the differential metabolites, including lipids, nucleotides, aldehydes, alcohols, ketones, alkaloids, flavonoids, and other organic compounds, among different cattle breeds, aiming to explore breed-specific flavor-related metabolomic pathways. Understanding the basis of the correlation between beef flavor profile and differential metabolites in breeds may help producers develop novel breeding strategies and provide scientific information for marketing preferences.

## 2 Methods and materials

### 2.1 Animals and sampling

From a commercial feedlot operated by Guizhou Cattle Industry Group Co. LTD (Anshun, China), nine cattle from three breeds (three bulls each), namely Guanling (GL), Sinan (SN), and Simmental crossbred (XZ, Simmental sires and Guanling dams), were randomly selected for this experiment. All animals were 30–32 months old and raised under the same conditions with forage-based finishing diets, which contained straw, corn, grain mixtures, and soybean meal, with a forage-to-concentrate ratio of 60:40 on a dry matter basis. Animals were fed twice daily at 08:00 and 16:00 with free access to water. The average finished body weight of GL, SN, and XZ were 477.9 kg, 387.6 kg, and 675.5 kg, respectively. Before slaughter, animals were transported to the abattoir one day earlier and subjected to 24 h of feed withdrawal and eight hours of water restriction. Standard commercial slaughter protocols were followed according to the Operating Procedure of Livestock and Poultry Slaughtering (GB/T 19477–2018). Briefly, animals were stunned by electrocution, followed by exsanguination through the jugular and carotid incisions. After evisceration was performed, carcasses were split into two halves (left and right). Then, samples of longissimus thoracis (LT) muscle, which is the representative muscle for conventional beef quality evaluation, were collected between the 12th and 13th ribs of each hot carcass. Immediately, all samples were vacuum-sealed in plastic bags to ensure sample integrity and then transported on ice to the lab for analysis.

All experimental procedures involving animals in this study received formal approval (AWE-GZSXMSY-2023-13) from the Institutional Animal Care and Use Committee at Guizhou Institute of Animal Husbandry and Veterinary Sciences.

### 2.2 Non-targeted metabolomics analysis (UHPLC-MS/MS)

Sample preparation was conducted following the method previously described (Warren et al., 2017). Briefly, 1.0 g of each meat sample was accurately weighed into 2 mL centrifuge tubes, and 1 mL of tissue extraction reagent (75 % 9:1 methanol: chloroform, 25 % H_2_O; Fisher Scientific, Loughborough, UK) was added. The mixture was homogenized at 50 Hz for 2 min using a tissue grinder, followed by ultrasonication for 30 min at room temperature and an additional 30 min in an ice bath. After centrifugation at 13,780 ×*g* for 10 min at 4 °C, the supernatant was collected, taken into a new tube, and vacuum freeze-dried. 200 μL of 50 % acetonitrile solution (Fisher Scientific, Loughborough, UK) containing 4 mg/L 2-Amino-3-(2-chlorophenyl)-propionic acid (Aladdin, Shanghai, China) were used to reconstitute the dried samples. Then, the dissolved samples were filtered by 0.22 μm membrane and transferred into detection vials for LC-MS analysis.

The untargeted metabolic profiling of beef meat was conducted using a Vanquish UHPLC System (Thermo Fisher Scientific, USA) coupled with an Orbitrap Exploris 120 mass spectrometer (Thermo Fisher Scientific, USA). The analysis method was adapted from the previous literature with some modifications (Zelena et al., 2009). Chromatography was achieved on a 2.1 × 100 mm ACQUITY UPLC HSS T3 column (1.8 μm, Waters, USA), which was maintained at 40 °C with a constant flow rate of 0.3 mL/min. For positive ion mode (ESI^+^) analysis, the mobile phases consisted of (solvent A) 0.1 % formic acid in water (*v*/v) and (solvent B) 0.1 % formic acid in acetonitrile (v/v). The gradient elution program was as follows: initial 8 % B (0–1 min), linear increase to 98 % B (1–8 min), held at 98 % B for two minutes (8–10 min), rapidly returned to 8 % B within 0.1 min (10–10.1 min) and re-equilibrated at 8 % B (10.1–12 min). Negative ion mode (ESI^−^) analysis employed (solvent A) 5 mM ammonium formate and (solvent B) acetonitrile with an identical gradient elution program.

Mass spectrometric detection was performed in full MS-ddMS^2^ mode with the following parameters: sheath gas pressure 40 arb, auxiliary gas flow 10 arb, dual spray voltages 3.50 kV for ESI^+^ and − 2.50 kV for ESI^−^, capillary temperature 325 °C. MS1 scanned from 100 *m*/*z* to 1000 m/z at 60000 FWHM (full width at half maximum) resolution. Data-dependent MS/MS acquisition selected four scans per cycle; MS/MS spectra were collected at 15000 FWHM with 30 % normalized collision energy and automatic dynamic exclusion time. (Want et al., 2013).

Metabolites identification of MS and MS/MS data was achieved by matching with public databases, including HMDB (Human Metabolome Database), Massbank, KEGG (Kyoto Encyclopedia of Genes and Genomes), LipidMaps, Mzcloud, and an in-house database (Panomix Biomedical Tech Co., Ltd., Suzhou, China). The molecular weight of metabolites was determined based on the mass-to-charge ratio of parent ions in the MS data. Molecular formulas were predicted by parts per million and adduct ion information, then matched against the database for metabolite identification. Additionally, MS/MS spectra from the quantitative data were compared with database records, such as fragment ion patterns, to confirm metabolite identities through MS/MS matching.

### 2.3 Two-dimensional gas chromatography time-of-flight mass spectrometry analysis (GC × GC-TOF MS)

The headspace solid-phase microextraction method (HS-SPME) was applied to extract volatile compounds. A 10 mg/L stock solution of n-Hexyl-d_13_ was prepared in 50 % ethanol and then stored at 4 °C until use as the internal standard for semi-quantification. Briefly, 2.0 g of samples were incubated with 10 μL of the internal standard solution at 80 °C for 10 min. A 50/30 μm divinylbenzene/carboxen/polydimethylsiloxane SPME fiber (Bellefonte, USA) was conditioned at 270 °C for 10 min prior to extraction. Then, the samples were extracted using the fiber at 80 °C for 40 min. The fiber was desorbed at 250 °C for 5 min in a GC injector. Post-injection, the fiber was reconditioned at 270 °C for 10 min. Saturated Alkanes (1 mg/L in N-Hexane; Sigma-Aldrich, USA) were used for the calibration of the retention index (RI). (Ruan et al., 2023).

GC × GC-TOF MS analysis was performed on a Pegasus 4D instrument (LECO, MI, USA) comprising an Agilent 8890 A gas chromatograph system (Agilent Technologies, CA, USA), which was featured with a split/splitless injector, dual-stage cryogenic modulator (LECO), and a TOFMS detector (LECO). The analysis method was referred to the previous literature with some modifications (Bueno et al., 2019) (Xiaoyu Wang et al., 2022). Chromatographic separation was achieved using a primary DB-Heavy Wax column (30 m × 250 μm Inner Diameter, 0.5 μm; Agilent, USA), with the following temperature program: initial hold at 50 °C for 2 min, then raised to 220 °C at 4 °C/min, and held for 13 min. The secondary Rxi-5Sil MS column (2.0 m × 150 μm I.D., 0.15 μm; Restek, USA) was maintained 5 °C above the primary oven temperature. High-purity helium (>99.999 %) carrier gas flowed at 1 mL/min. The modulator operated with a 5.0 s modulation period and a 15 °C offset above the secondary column temperature. The injection temperature of GC was 250 °C. Mass spectrometric detection of volatile compounds employed electron ionization (70 eV) with a scan range of *m*/*z* 35–550. The ion source and transfer line temperatures were set to 250 °C, with a detector voltage of 2031 V. Data acquisition occurred at 200 spectra/s.

Raw data was analyzed and processed using Chroma TOF software (version 4.50). The thresholds were set as follows: Signal-to-Noise (S/N) ratio ≥ 50 and similarities ≥700. The recorded mass spectra were cross-checked against the NIST spectral library to qualitatively and quantitatively identify the volatile organic compounds. The difference between the theoretical RI and the calculated RI was set to be less than 20 to ensure reliable confirmation of the compound identity and avoid abused confirmation. Internal standard normalization was performed on the raw data. Compounds with more than 50 % missing data were excluded from further analysis. The classification of VOCs was based on PubChem database. VOC-sensory attribute networks were constructed using the R package igraph based on FlavorDB database.

The contributions of individual VOCs to the overall aroma profile were assessed via the calculation (0–100 scale) of Relative Odor Activity Value (ROAV) for each compound. It was calculated as follows: ROAV_i_ = (OAV_i_/OAV_max_) × 100, where OAV_max_ denotes the highest odor activity value (OAV) among all detected volatile compounds and OAV_i_ represents the odor activity value of a specific volatile compound. The calculation formula of a volatile compound's OAV is OAV_i_ = C_i_/OT_i_, where C_i_ means the relative content of the compound and OT_i_ is its odor threshold in water (Zhu et al., 2020).

### 2.4 Data analysis

Principal component analysis (PCA) and orthogonal partial least-square discriminant analysis (OPLS-DA) were applied to determine the distinction of beef metabolites among different breeds by OriginLab 2018 software (OriginLab Corporation, Northampton, MA, USA).

Pathway analysis of differential metabolites between cattle breeds was conducted by MetaboAnalyst 5.0, where the KEGG pathway was used for functional annotation (https://www.metaboanalyst.ca/MetaboAnalyst/ModuleView.xhtml). Visualization of the metabolites and corresponding pathways was constructed using the KEGG Mapper tool.

The Pairwise Spearman Rank correlation analysis was carried out on the differential VOCs and non-volatile metabolites. The heatmap visualization was constructed using the Double Matrices Correlation module in ChiPlot (a comprehensive web service for data analysis and visualization, https://www.chiplot.online).

Differences between groups were assessed for significance using a one-way analysis of variance (ANOVA). The false discovery rate (FDR) was applied to adjust the *p*-value using Benjamini and Hochberg's approach. Statistical significance was defined as an adjusted *p*-value <0.05.

## 3 Results and discussion

### 3.1 UHPLC-MS/MS analysis

#### 3.1.1 Metabolic profiling revealed differences among breeds

Both positive (ESI^+^) and negative (ESI^−^) ion modes of the untargeted UHPLC-MS metabolomics analysis were conducted to fully detect metabolites in beef. The consistent response strengths and retention time of the total ion chromatograms (TICs) of the quality control samples across both ion modes proved the stability of the instruments and operations and the reliability of the obtained data (Fig. S1).

**Fig. 1:**
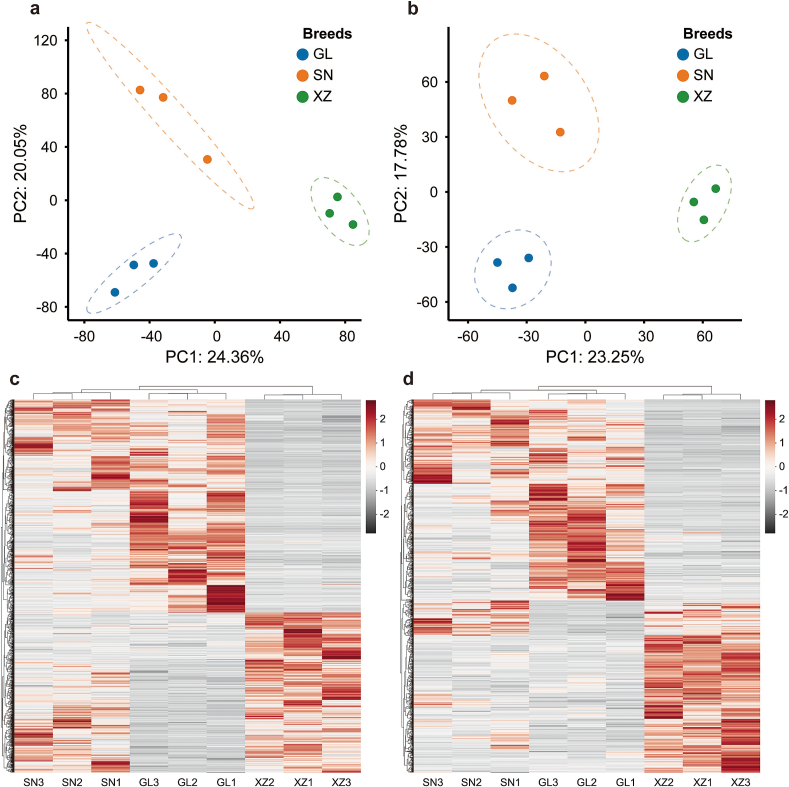
Non-targeted metabolomics analysis of the beef from three cattle breeds. Principal component analysis (PCA) of metabolites identified by LC-MS in (a) positive ion mode and (b) negative ion mode; all plots with 95 % confidence ellipsoids. Hierarchical cluster analysis of metabolites in beef of different breeds identified in (c) positive ion mode and (d) negative ion mode; Euclidean distance method with complete linkage was applied; each column represents one sample, and each row represents one metabolite; red indicates high abundance, grey indicates relatively low abundance (z-score normalization). (For interpretation of the references to colour in this figure legend, the reader is referred to the web version of this article.)

After peak-picking and alignment, a total of 13,038 metabolites were detected in positive ion mode, and 8333 metabolites were detected in negative ion mode. PCA and OPLS-DA were performed to evaluate the maximum variance among metabolite profiles. PCA explained 24.36 % (PC1) and 20.05 % (PC2) of the total variation in the positive ion mode (Fig. 1a), and 23.25 % (PC1) and 17.78 % (PC2) in the negative ion mode (Fig. 1b). As a result, different cattle breeds were appropriately distinguished, implying distinct metabolite profiles among the breeds.

In addition, the OPLS-DA model also exhibited a clear separation of metabolites among cattle breeds (Fig. S2). The explanatory power and predictive performance were R^2^Y = 0.998 and Q^2^ = 0.84 in the positive mode and R^2^Y = 0.999 and Q^2^ = 0.741 in the negative mode, which suggested that the model was feasible for further screening of markers of differences. Theoretically, R^2^ and Q^2^ values approaching 1 are ideal. Generally, values above 0.5 are required, while those exceeding 0.4 are considered acceptable (Yun et al., 2021). Subsequently, the identification of differential metabolites was achieved by the combination criteria of VIP > 1 and *p* < 0.05. In positive ion mode, 1798, 3079, and 2368 differential metabolites were identified in the comparison of SN vs. GL, GL vs. XZ, and SN vs. XZ, respectively. In negative ion mode, the comparison of SN vs. GL, GL vs. XZ, and SN vs. XZ revealed 961, 1644, and 1402 differential metabolites, respectively (Fig. S3). Generally, the difference in beef metabolites between Guizhou yellow cattle breeds and the Simmental crossbreed was greater than the difference within Guizhou yellow cattle breeds, which was also revealed by the heatmap plot of metabolites generated by hierarchical clustering analysis (Fig. 1c,d).

#### 3.1.2 Differential metabolites identification and classification

**Fig. 2:**
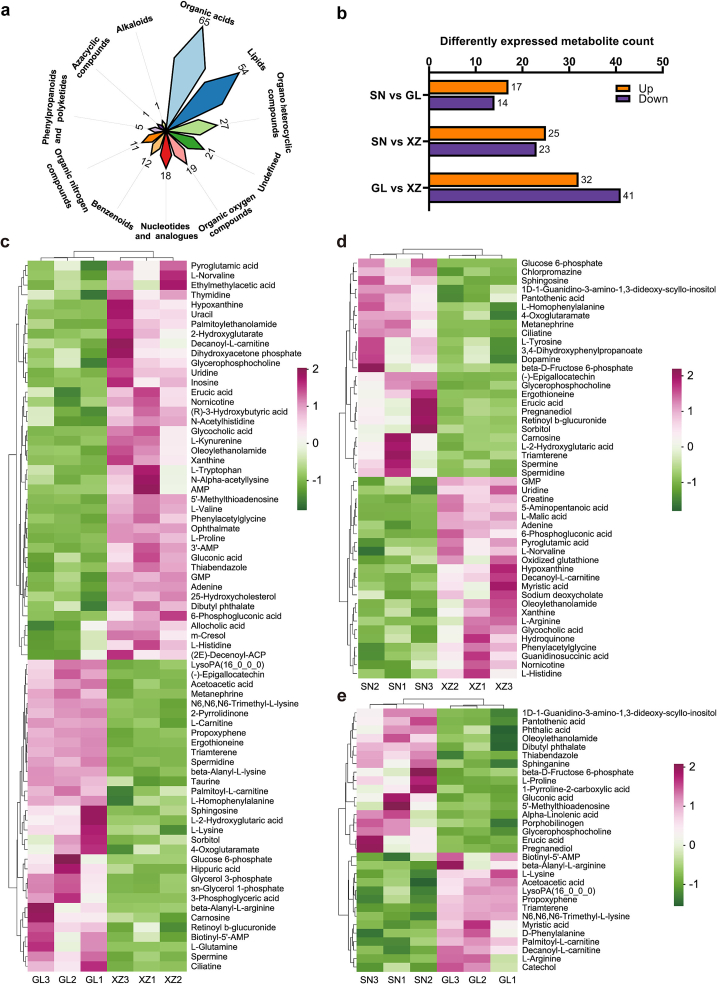
Differential metabolites analysis of beef among different breeds identified by MS-MS. (a) Rose plot of the composition classes of the differential metabolites. (b) The numbers of up-regulated (orange) and down-regulated (purple) metabolites between each two experimental groups (*p* < 0.05). Heatmap of the levels of differential metabolites between (c) GL and XZ, (d) SN and XZ, and (e) GL and SN. The name of each metabolite is provided in each row of the heatmaps. Euclidean distance method with complete linkage was applied. Red indicates up-regulation; green indicates down-regulation (z-score normalization). (For interpretation of the references to colour in this figure legend, the reader is referred to the web version of this article.)

A total of 2810 differential metabolites were annotated in positive ion mode, while 2035 differential metabolites were annotated in negative ion mode. Subsequently, all these metabolites were integrated to match against the databases using their retention time and the secondary mass spectra, which included all fragment ions of the substances. As a result, 234 non-volatile metabolites were identified for further analysis. These metabolites consisted of various biochemical classes, including 65 organic acids, 54 lipids, 27 organic heterocyclic compounds, 18 nucleotides and analogues, 19 organic oxygen compounds, 12 benzenoids, 11 organic nitrogen compounds, five phenylpropanoids and polyketides, one alkaloid, one azacyclic compound, and 21 undefined metabolites (Fig. 2a). In the comparison of SN vs. GL, 17 up-regulated and 14 downregulated metabolites were observed. There were 32 up-regulated metabolites and 41 downregulated metabolites in the comparison of GL vs XZ. Compared to XZ, SN had 25 up-regulated and 23 downregulated metabolites (Fig. 2b). The heat map (Fig. 2c–e) displayed the relative abundance of these metabolites, which may be representatives of the differential metabolites among cattle breeds. Notably, more pronounced differences were observed between GL and XZ. This divergence could be attributed to their genetic background, as the origins of Guanling and Simmental cattle differed markedly (Xu et al., 2024). Comparative studies on Hanwoo and Angus cattle demonstrated that breed has significant effects on sensory characteristics and volatile flavor compounds, likely due to the difference in lipid-derived metabolite content between the breeds (Van Ba et al., 2013).

#### 3.1.3 Metabolite variations across diverse metabolic pathways

**Fig. 3:**
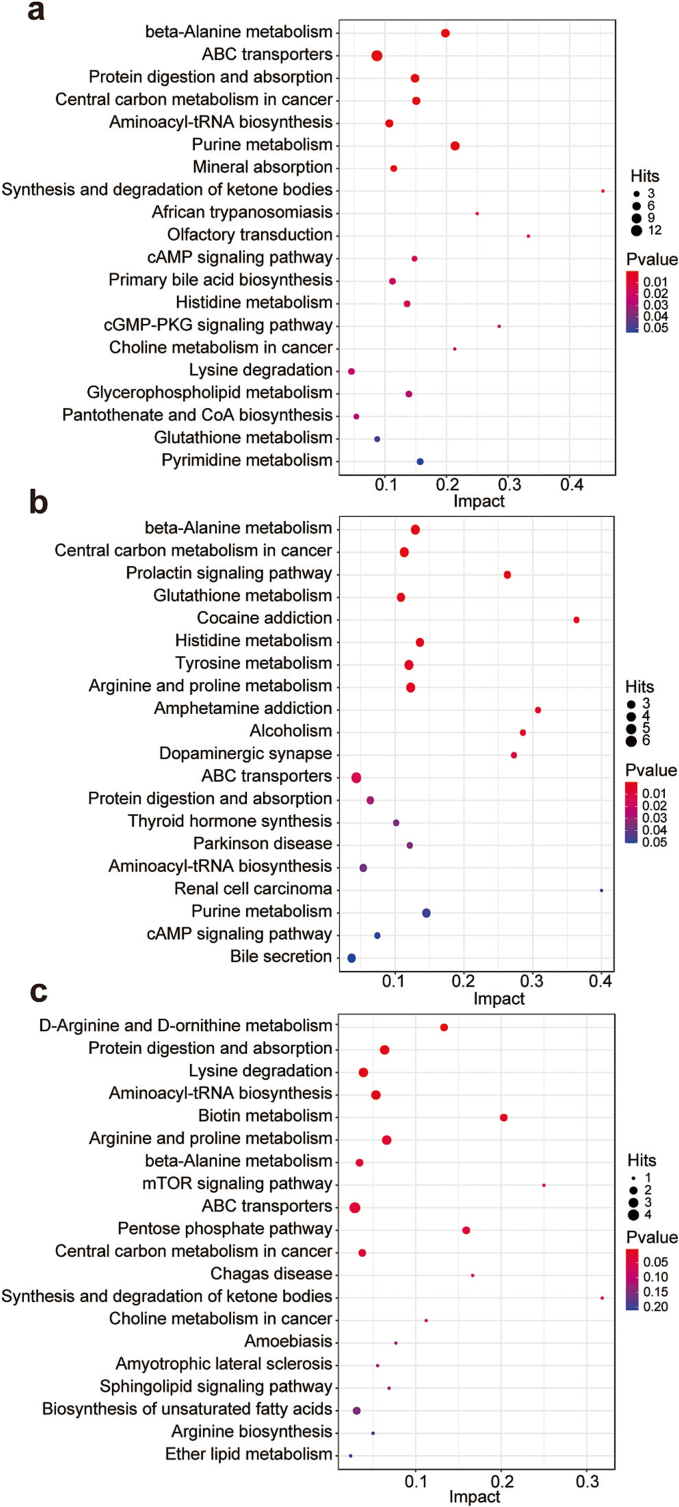
Metabolite pathway enrichment analysis plot of metabolite biomarkers. Kyoto encyclopedia of genes and genomes (KEGG) pathway analysis displays the enrichment and association map of the differentially expressed metabolites between (a) GL and XZ, (b) SN and XZ, and (c) GL and SN.

To investigate the functions and outcomes of these differential metabolites and the direct connections of the metabolites in biochemical reaction networks (Klamt & Stelling, 2003), we conducted pathway analysis of the significantly altered metabolites. The top 20 enriched metabolic pathways between breeds are presented in Fig. 3. Herein, we focused on pathways relevant to meat flavor despite other crucial differences in various pathways among cattle breeds. The differential metabolites of GL and XZ were assigned to beta-alanine metabolism, purine metabolism, protein digestion and absorption, synthesis and degradation of ketone bodies, olfactory transduction, histidine metabolism, lysine degradation, glutathione metabolism, glycerophospholipid metabolism, and pantothenate and CoA biosynthesis. The important metabolic pathways identified for the comparison of SN and XZ were beta-alanine metabolism, glutathione metabolism, histidine metabolism, tyrosine metabolism, arginine and proline metabolism, protein digestion and absorption, and purine metabolism. Only 11 metabolic pathways were significantly influenced for the comparison of SN and GL (*p* < 0.05), including d-arginine and d-ornithine metabolism, protein digestion and absorption, lysine degradation, arginine and proline metabolism, and beta-alanine metabolism. Alterations in these metabolic pathways are crucial to the unique flavor and taste of different beef breeds.

#### 3.1.4 Key metabolic pathways associated with flavor development

**Fig. 4:**
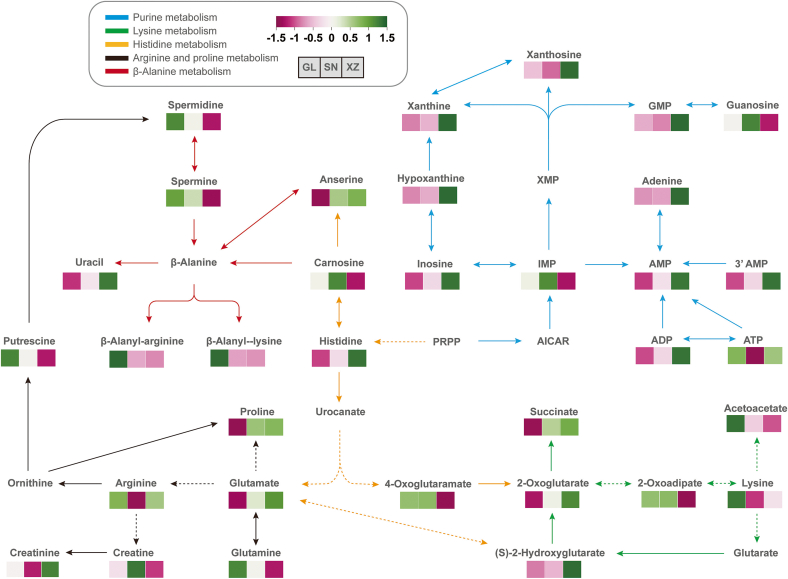
The integrated metabolic networks of differential metabolites and related metabolism pathways contributed to the difference in beef flavor among three breeds of cattle. Relative abundances of the metabolites are shown in the heatmap; red denotes up-regulation, and green denotes down-regulation. Pathways are accordingly presented in different colors of arrows. (For interpretation of the references to colour in this figure legend, the reader is referred to the web version of this article.)

There are many flavor precursors in raw meats, such as amino acids, organic acids, nucleotides, and sugars. The stimulation of these precursors by heat during cooking led to the development of the final flavor (Shahidi & Hossain, 2022). The enriched metabolic pathways were primarily highlighted by the difference in amino acids (Fig. 4). Beta-alanine, a naturally occurring β-type amino acid, has been widely used in food industries (L. Wang et al., 2021). When β-alanine and L-histidine are bonded, a flavor precursor, carnosine, is synthesized (Blancquaert et al., 2016). This umami taste histidine-containing dipeptides is rich in livestock animals, such as beef cattle, chicken, and pigs (Ge et al., 2023; Kim et al., 2024). The addition of carnosine into the reaction mixtures generally inhibited the formation of sulfur-containing compounds, including thiophenes, 2-furfurylthiol, 2-methyl-3-furanthiol, and their associated dimers, which are critical for meaty flavor. Simultaneously, carnosine promoted the generation of key roasted and nutty flavor compounds, such as thiazoles and pyrazine along with its alkyl and methyl derivatives (Y. Chen & Ho, 2002). Moreover, higher levels of peptides containing alanyl residues, such as beta-alanyl-L-arginine and beta-alanyl-l-lysine, may also be associated with the umami taste of Guanling cattle.

In pathways like arginine, proline, and lysine metabolism, many flavor substances are also involved (Fig. 4). Glutamate and glutamine are the most important amino acids associated with savory taste and umami flavor. Proline elicits a sweet-bitter taste, while arginine has a bitter taste (Birch & Kemp, 1989). In fact, D-amino acids primarily possessed a sweet flavor, with the exception of proline and hydroxyproline, while seven of the L-amino acids also taste sweet (Birch & Kemp, 1989). These amino acids have been evidenced to activate the taste receptor, enabling the detection of savory or delicious flavors (Nelson et al., 2002).

Through the breakdown of arginine, biogenic amines like ornithine and putrescine can be generated (Fig. 4), which can influence the overall taste and aroma by contributing to the gamey or fermented flavor (Ashaolu et al., 2023). Putrescine is the precursor of spermidine, a polyamine critically involved in regulating glutathione metabolism (Fig. 4). Specifically, in response to oxidative stress conditions, the enzyme activity and decomposition of glutathione were suppressed by spermidine, resulting in the enhancement of cellular protection against oxidative stress and inflammatory damage with more glutathione available (Teng et al., 2022). Biogenic amines are naturally present in a wide range of meats, particularly abundant in beef, mutton, chicken, and fish (Schirone et al., 2022). Among various biogenic amines, the structurally analogous polyamines spermine and spermidine have often been used as practical biomarkers for assessing meat product quality (Triki et al., 2018). The levels of spermine tended to be highest in fresh meat, including red meat (Holbert et al., 2022). However, high concentrations of biogenic amines are also considered an indication of food spoilage.

While glutathione metabolism is well known to be involved in antioxidant processes, it also contributes to the flavor development process in meats (Setyabrata et al., 2021). As electrophysiologically verified by taste nerve responses, glutathione (γ-glutamyl-cysteinyl-glycine) demonstrated strong molecular interactions with umami compounds, thereby influencing flavor development (Yamamoto & Inui-Yamamoto, 2023). Moreover, glutathione exhibited a distinctive kokumi flavor, which is characterized by continuity, mouthfulness, and thickness (Ueda et al., 1997). This compound was identified as a potent calcium-sensing receptor (CaSR) agonist, demonstrating significant kokumi-enhancing properties in chicken broth. Furthermore, when introduced individually to aqueous solutions of sucrose, NaCl, or Monosodium Glutamate (MSG), glutathione selectively enhanced the perception of sweet, salty, and umami tastes (Ohsu et al., 2010).

Purine metabolism was found to be closely associated with meat quality and flavor (Ge et al., 2023). Although the relationship between purine and the sensory characteristics of beef is not well understood, it is known that high-purine foods exhibit elevated umami intensity in general (Johnson et al., 2013). There is also a link between high purine content in food consumption and the incidence of gout and obesity (Andres-Hernando et al., 2021). In the current study, IMP, AMP, and GMP were identified as differential metabolites, along with other purine bases such as xanthine, hypoxanthine, adenine, and l-glutamine, all of which are involved in the purine metabolic pathway (Fig. 4). Most purine alkaloids, such as derivatives of xanthine, taste bitter to humans. Lower levels of inosine, xanthine, xanthosine, and hypoxanthine were found in GL and SN compared to XZ, suggesting their meat flavor was less bitter. The umami taste sensation was amplified up to 8-fold by the synergistic effect of glutamate and flavor enhancers such as IMP, AMP, and GMP (Ninomiya, 1998). Compared to XZ, GL and SN possessed higher levels of IMP and lower levels of GMP and AMP (Fig. 4). Additionally, GMP, IMP, and AMP are known to be umami and sweet taste transduction by membrane depolarization and potassium permeability modulation (Tonosaki & Funakoshi, 1988). However, a weak inherent taste intensity of nucleotides was established (Yamaguchi & Ninomiya, 2000).

### 3.2 GC × GC-TOF MS analysis

#### 3.2.1 Beef aroma profiling among breeds and volatile compounds screening

**Fig. 5:**
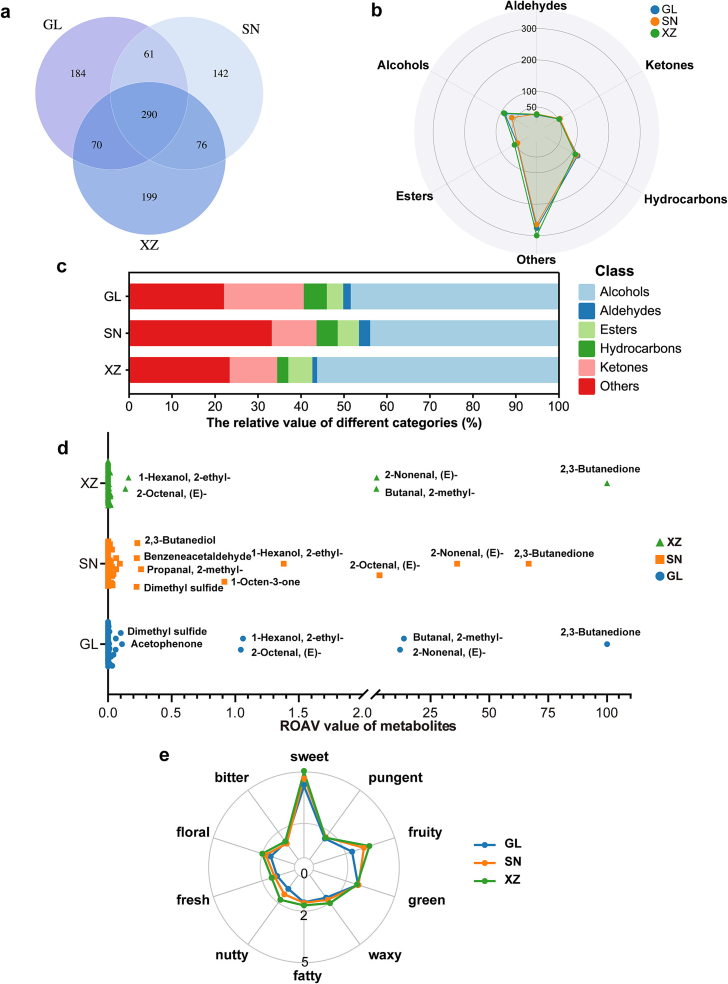
GC × GC-TOF MS analysis of the beef from three cattle breeds. (a) Venn diagram of the numbers of VOCs in three groups. (b) Numbers of VOCs classes based on chemical taxonomy. (c) Proportion of VOCs in each group. (d) ROAV analysis of the contribution of VOCs to aroma. Compounds with ROAV >0.1 are labeled. (e) Sensory profile evaluation of VOCs obtained from beef of three breeds.

Following the characterization of meat taste by identifying differential metabolites among breeds, we exerted effort toward the investigation of specific flavor volatiles to define the meat aroma profile of Guanling, Sinan, and Simmental crossbred cattle. Here, GC × GC-TOF MS analysis was conducted, resulting in apparent peak separations and high stability of data (Fig. S4). A total of 1022 volatile compounds (VOCs) with CAS annotations were identified, including hydrocarbons, alcohols, aldehydes, esters, ketones, and others (such as organic heterocyclic compounds, lipids, and benzenoids) (Fig. 5a,b). Guanling cattle exhibited a total of 605 VOCs, with 184 being unique to this breed. Sinan cattle had 569 VOCs, including 142 unique ones. On the other hand, 199 unique VOCs were found in the Simmental crossbreed from a total of 635 VOCs identified. Across all three breeds, 290 types of volatile compounds were commonly shared. A prominent disparity in alcohol-type VOCs was highlighted among the cattle breeds, with SN exhibiting lower diversity (62 types) compared to GL and XZ, which contained 88 and 91 alcohols, respectively (Fig. 5b). This may reflect variations in breed-specific biochemical pathways influencing VOC production.

In terms of the composition of VOCs, the relative contents of alcohols and ketones in GL were 48.42 % and 18.58 %, respectively (Fig. 5c). Ketones primarily result from Maillard reaction, lipid oxidative degradation, and thermal modification of amino acids, commonly contributing oil and floral aromas, which are very significant for beef taste (Xiaodan Wang et al., 2018). Alcohols and other volatile compounds accounted for 77.11 % of the total amount of VOCs in SN. Similarly, alcohols and other volatile compounds dominated in XZ with 56.25 % and 23.41 % of the relative content, respectively (Fig. 5c). Alcohols primarily originate from the free-radical-induced decomposition of saccharides due to lipid oxidation and are considered key fragrant substances. Although alcohols have been considered to contribute less to the overall flavors in beef due to the relatively high odor thresholds, certain alcohols serve as crucial intermediates in the synthesis of heterocyclic compounds, thereby playing a key role as substrates in the development of beef flavors (Zang et al., 2020).

To further characterize the contribution of VOCs to the aroma of beef, the relative odor activity values (ROAV) were assessed herein. When a ROAV value exceeds one, it typically indicates that the volatile compound plays an important role in the aroma. In contrast, compounds with values ranging from 0.1 to 1 are considered to have a moderate degree of influence on the aroma (Li et al., 2023). All compounds with ROAV larger than 0.1 are shown in Fig. 5d and Table S1. 2,3-Butanedione with pleasant buttery odors contributed the most to the aroma of all three breeds. 2,3-Butanedione was reported to be a key aroma compound in raw and cooked dzo beef (Wan et al., 2023) and has the highest ROAV value in most animals' milk (Sun et al., 2023). In a study comparing flavor substances in air-dried beef from different regions of Inner Mongolia, 2,3-Butanedione (ROAV >1) was found to be a unique flavor substance in certain regions. Additionally, vacuum tumbling of air-dried beef had an enhancing effect on the ROAV of 2,3-Butanedione when compared to the static curing method (Shiqi et al., 2024). Furthermore, the ROAVs of 2-methyl-butanal, (E)-2-nonenal, 2-ethyl-1-hexanol, (E)-2-octenal, acetophenone, and dimethyl sulfide in GL were larger than one, providing significant “almond”, “fatty”, “cucumber”, “rose”, “green”, and “nuts” aromas. Moreover, SN exhibited high abundances of “fatty”, “cucumber”, and “green” aromas as the ROAVs of (E)-2-nonenal, (E)-2-octenal, 2-ethyl-1-hexanol were larger than one. Similarly, (E)-2-nonenal was found to be a key flavor compound that contributes to “green” flavor characteristics in Xinjiang brown beef (Ma et al., 2024). (E)-2-octenal and 2-methyl-butanal were detected in XZ with ROAV >2, giving rise to a greater prevalence of “fatty”, “cucumber”, “cocoa”, and “almond” aromas. Overall, XZ demonstrated enhanced flavor profiles in most aromas notes compared to SN and GL (Fig. 5e). However, aroma omission experiments have suggested that ROAV alone cannot determine the impact of aroma compounds on the overall aroma profile, which was influenced by complex interactions between compounds, such as masking, antagonism, and synergy (X. Chen et al., 2024).

#### 3.2.2 Multivariate analysis revealed differences among volatile compounds profiles

OPLS-DA was carried out to reveal the difference in beef samples by providing a deep analysis of the main matrix characteristics of the volatile compounds. Firstly, the OPLS-DA model with R^2^Y = 0.989 and Q^2^ = 0.495 showed a clear separation of breeds on PC1 (17.2 %) and a spread of samples within each group (15.8 %) (Fig. S5a). These results suggested that the aroma profiles of beef meats varied among breeds (Fig. 5e). Secondly, differentially expressed VOCs were screened out using VIP > 1 and *p* < 0.05 as thresholds to further explore the difference in flavor between breeds. When compared to XZ, GL had two higher levels and 13 lower levels of compounds (Fig. S5b). The abundance of six compounds increased, and 10 decreased in SN meat compared with XZ (Fig. S5c). Two compounds were up-regulated, and 10 were down-regulated in the comparison between GL and SN (Fig. S5d).

**Fig. 6:**
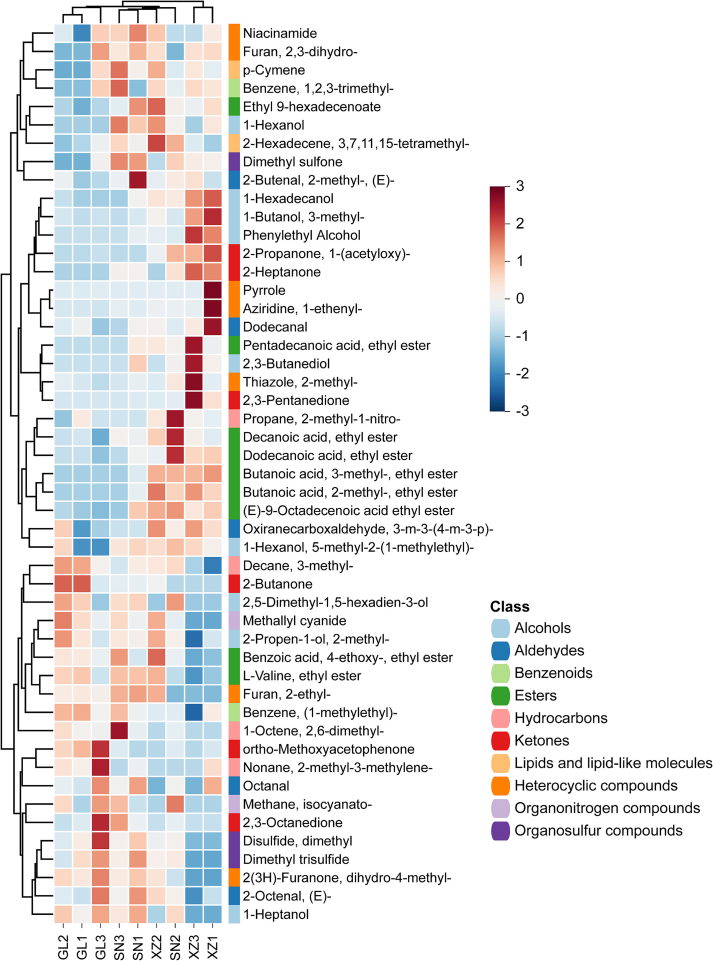
Hierarchical cluster analysis of differential VOCs in beef of three breeds. Euclidean distance method with complete linkage was applied. Red indicates up-regulation; blue indicates down-regulation (z-score normalization). Significant differential VOCs are screened by OPLS-DA aided with VIP >1 and *p* < 0.05. (For interpretation of the references to colour in this figure legend, the reader is referred to the web version of this article.)

The contents of differential VOCs among three breeds of beef meat were presented in the heat map (Fig. 6), which shows their differences mainly originated from variations in alcohols, esters, ketones, and heterocyclic compounds. Generally, the volatile compound compositions of GL and SN were similar, while XZ exhibited greater divergence from the local yellow cattle breeds. However, these differences arose from different sets of VOC classes. For SN and XZ, the differences mainly came from the variations in alcohol substances. In contrast, lower esters and ketones content in GL resulted in a distinct aroma profile compared to SN and XZ. Esters are generated from the reaction of alcohols and carboxylic acid derivatives and feature a wide range of fruity or buttery flavors (Forney & Song, 2017). Some esters have been used as food additives to neutralize the unpleasant odor of meat products and impair desired flavors (Dong et al., 2024).

#### 3.2.3 Network interactions of VOCs and sensory aroma characteristics

**Fig. 7:**
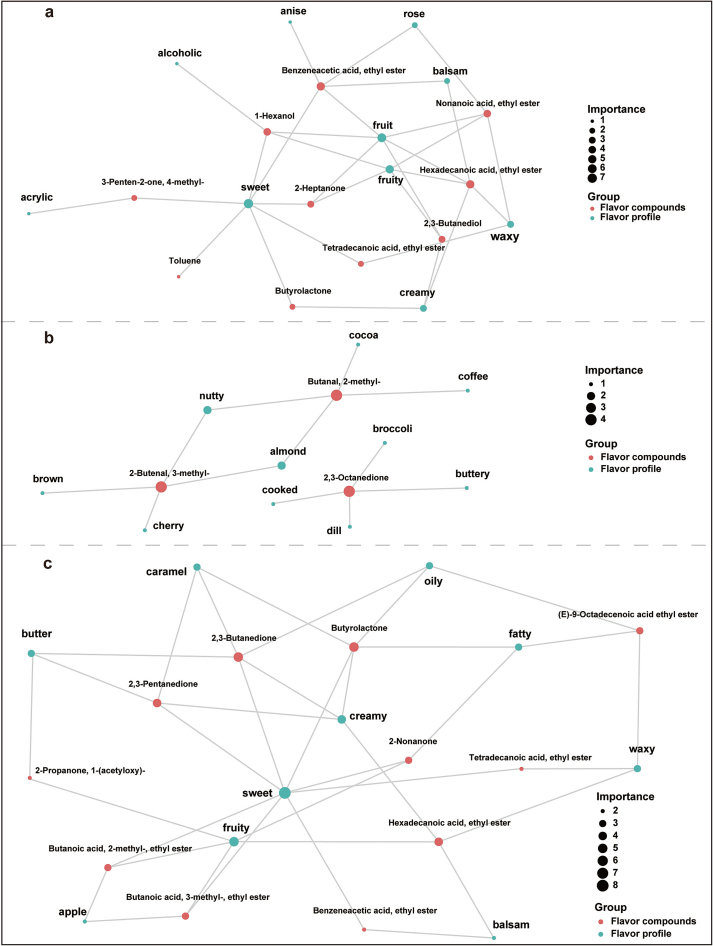
The network diagram of the association between differential VOCs and sensory flavor attributes. (a) Comparison of GL and SN; (b) comparison of SN and XZ; (c) comparison of GL and XZ. Larger blue circles denote sensory attributes connected by more VOCs; larger red circles denote VOCs contributing to more sensory attributes. Only the top 10 sensory attributes are used for the network diagram. (For interpretation of the references to colour in this figure legend, the reader is referred to the web version of this article.)

The interaction network between VOCs and sensory aroma characteristics was visualized by using FlavorDB database and igraph tools. Fig. 7 demonstrated that the significant VOCs contributed to the 10 most important aromas, such as “sweet”, “fruity”, “green”, “waxy”, “ethereal”, and “floral”. It is clear that one type of aroma is modulated by multiple compounds, one of which also contributes to multiple aroma characteristics. Therefore, changes in the abundance of one compound may influence the aroma profile of beef. Nevertheless, it is worth noting that the effect of this change on the aroma characteristic could be neutralized or compensated by other compounds.

### 3.3 Correlation analysis of VOCs and non-volatile metabolites

**Fig. 8:**
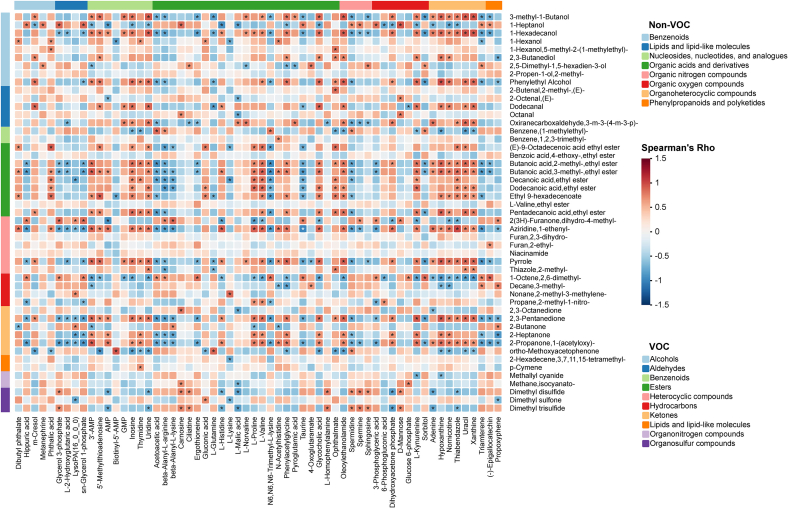
Correlation analysis between differential metabolites and VOCs. Heatmap colors indicate Spearman's Rho; red and blue indicate positive and negative correlation, respectively. Non-volatile metabolites are listed on the rows (right) and VOCs are listed on the column (bottom). Asterisks represent significant correlations with *p* < 0.05. (For interpretation of the references to colour in this figure legend, the reader is referred to the web version of this article.)

To better understand the formation of unique flavors in different beef breeds, a correlation analysis that elucidates the relationship between the metabolome of LC-MS (non-VOCs) and GC × GC-TOF MS (VOCs) was performed (Fig. 8). Overall, volatile compounds, including alcohols, esters, ketones, and some heterocyclic compounds, showed significant positive or negative correlations with various non-volatile metabolites. These correlations suggest that the formation of these VOCs relied on the presence of metabolic precursors. In the present study, a notable number of VOCs were accurately identified by GC × GC-TOF MS, while the majority of VOCs were derived from the catabolism of upstream metabolites. This is due to the inherent lack of enzymatic machinery for secondary metabolism in animals (Ramalingam et al., 2019). Flavor precursors undergo various processes, such as deamination, enolization, decarboxylation, and degradation, to produce furans, glycosyl aldehydes, amino ketones, keto-aldehydes, and other compounds (Ramalingam et al., 2019). Non-volatile compounds such as inosine, uridine, thymidine, 3’AMP, L-proline, L-valine, ophthalmate, oleoylethanolamide, L-kynurenine, hypoxanthine, nornicotine, thiabendazole, uracil, and xanthine were significantly correlated with many VOCs, mostly in a positive way. In contrast, negative correlations with VOCs were predominantly observed in acetoacetic acid, beta-alanyl-L-arginine, beta-alanyl-l-lysine, N6,N6,N6-trimethyl-l-lysine, taurine, spermidine, triamterene and aldehyde metabolites. These results suggested that these non-volatile metabolites may be important flavor precursors, the contents of which are key factors of flavor development in beef. Many of these flavor compounds and intermediates have their own distinct flavors. Meanwhile, the rich source of these intermediates also enables further reactions in the presence of ammonia, hydrogen sulfide, amino acids, aldehydes, alcohols, and other compounds, ultimately generating a cascade of flavor substances such as pyridines, pyrazines, imidazoles, pyrroles, thiazines, furans, ketones, and aldehydes (Arshad et al., 2018).

## 4 Conclusion

This study systematically compared the flavor profiles of beef from Guanling (GL), Sinan (SN), and Simmental crossbred (XZ) cattle using an integrated flavoromics strategy, revealing substantial interbreed variations in non-volatile metabolites and volatile organic compounds (VOCs). Based on the numbers of differential metabolites and VOCs, the differences within Guizhou yellow cattle breeds (SN vs GL) were relatively minor, whereas larger distinctions were observed between Guizhou yellow cattle and the Simmental crossbreed. Although XZ exhibited greater VOC diversity, GL cattle demonstrated a more distinct VOC composition and aroma profile compared to SN and XZ. This result may be explained by the different genetic distances between breeds, which requires further validation by incorporating multi-omics approaches (e.g., genomics, transcriptomics) to establish causal relationships between gene regulation and flavor metabolite production. Alcohols, ketones, heterocyclic compounds, and esters were the predominant differences, leading to aroma profiles that differed in “sweet”, “fruity”, “green”, “waxy”, “ethereal”, and “floral” characteristics among breeds. Breed-specific aroma profiles may be attributed to metabolic differences, particularly in amino acids and lipids pathways, with strong metabolite-VOC correlations supporting these findings. The limited sample size may have reduced the sensitivity to detect the differences among breeds, particularly for those subtle effects. Although the focus of this study is the significant variations detected within this sample size, increasing the sample size in future studies for a higher statistical power could enhance the ability to detect small and specific effects. Another limitation of this study is the restricted sample scope, which included only three cattle breeds. Future research could expand breed comparisons by incorporating purebred Simmental samples to help distinguish the effects of crossbreeding from innate breed-specific metabolic traits. Collectively, these findings provide a fundamental understanding of how breed influences flavor and offer insights into strategies for breeding cattle to improve flavor traits. Future studies could refine precision breeding strategies to optimize beef quality while maintaining productivity, ultimately benefiting both the meat industry and consumers.

## CRediT authorship contribution statement

**Yuanfeng Zhao:** Writing – review & editing, Writing – original draft, Visualization, Formal analysis, Conceptualization. **Jingrui Zhou:** Writing – review & editing, Investigation, Data curation, Conceptualization. **Wenju Luo:** Investigation. **Lingling Jiang:** Resources, Methodology. **Fuzhan Song:** Formal analysis. **Jiang Ran:** Validation, Resources. **Haoxiang Xu:** Methodology. **Lu Lei:** Investigation. **Rong Ai:** Validation. **Bo Yu:** Writing – review & editing, Supervision, Funding acquisition.

## Funding

This research was supported by the 10.13039/501100010909Young Scientists Fund of Guizhou Academy of Agricultural Sciences ([2023] No.04), the Science and Technology Support Program of Guizhou Province (Research Project No. QKHZC-2021-132), the Science and Technology Major Special Project of Guizhou Province (Research Project No. QKHZDZXZ-2020-3009-4), and the Earmarked Fund for Guizhou Modern Agriculture Research System (GZRNCYJSTX-05).

## Declaration of competing interest

The authors declare that they have no known competing financial interests or personal relationships that could have appeared to influence the work reported in this paper.

## Data Availability

Data will be made available on request.

## Appendix A Supplementary data

Supplementary figures and tables Image 1
