# Supplementary material for: An integrated metabolomics-flavoromics analysis reveals flavor diversity in indigenous and hybrid cattle via UHPLC-MS/MS and GC×GC-TOF-MS
Source: Food Chem X. 2025 Jul 16;29:102801. doi: 10.1016/j.fochx.2025.102801 (PMC12302928; doi:10.1016/j.fochx.2025.102801)
Supplement: Supplementary file 1 — Supplementary figures and tables [file mmc1.docx]

**Supplementary materials**


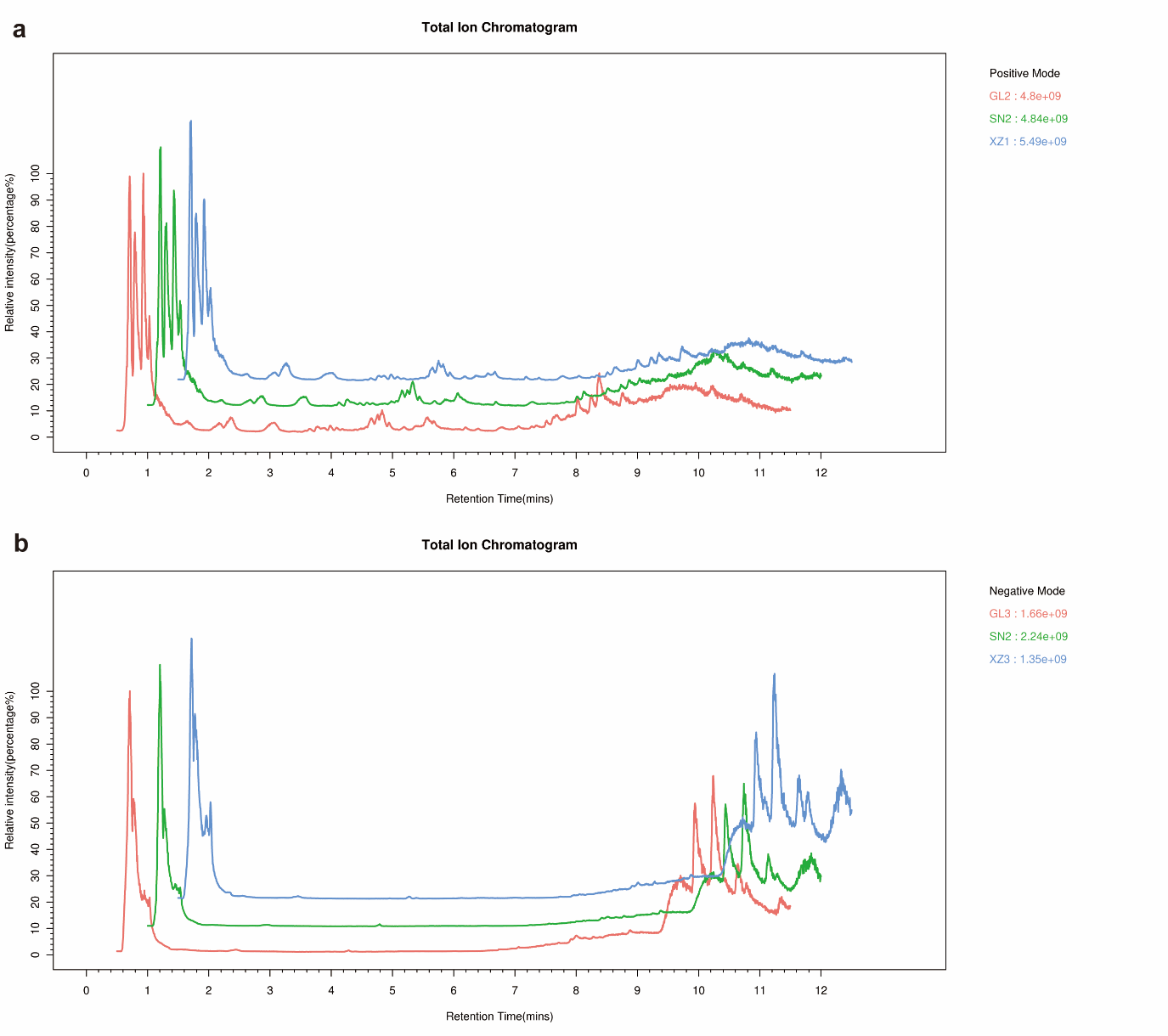


**Fig. S1.** The total ion chromatograms of quality control samples in positive ion mode (a) and negative ion mode (b) analyzed by UHPLC-MS.


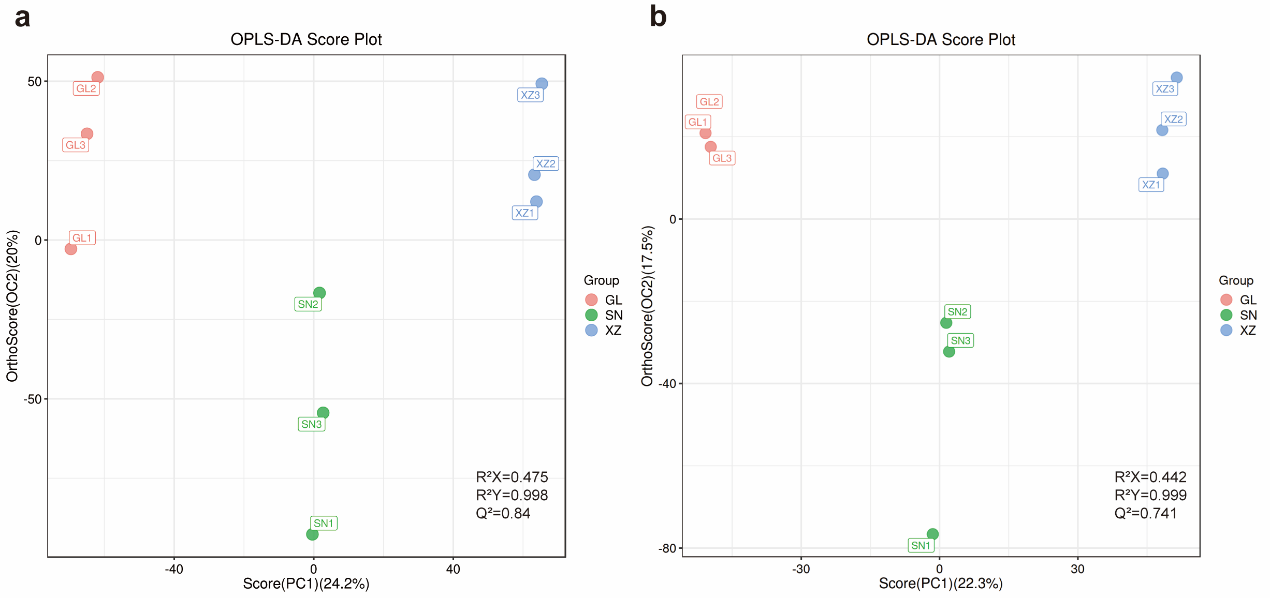


**Fig. S2.** The OPLS-DA score plots of identified metabolites of different groups in positive mode (a) and negative ion mode (b).


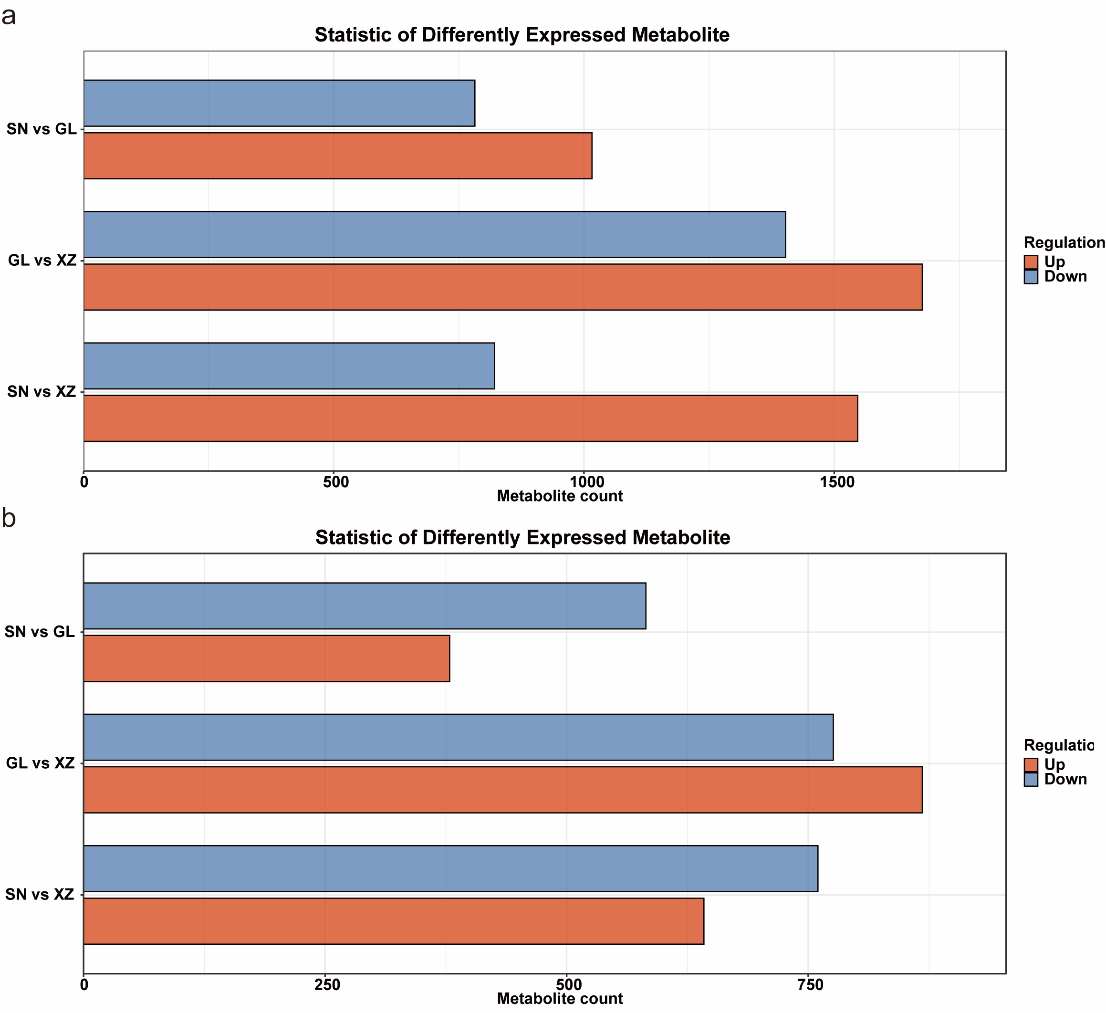


**Fig. S3.** Statistics on the change of differently expressed metabolites among the three breeds of beef (P < 0.05) in positive ion mode (a) and negative ion mode (b). Up-regulation shown in red, down-regulation shown in blue.


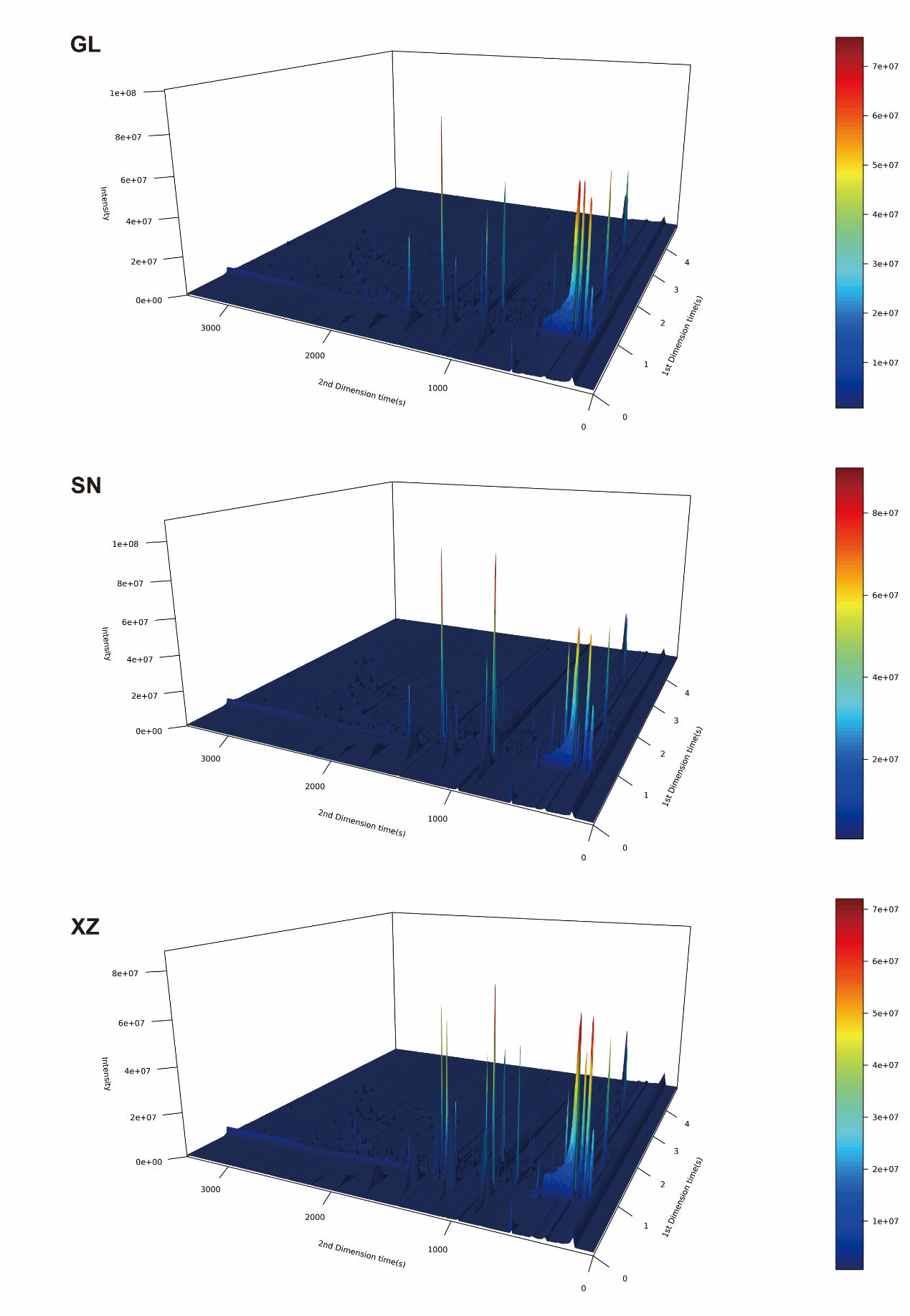


**Fig. S4.** Chromatographic 3D plots of the representative beef samples and VOCs (volatile organic compounds) identified in them using GC × GC-TOF MS.


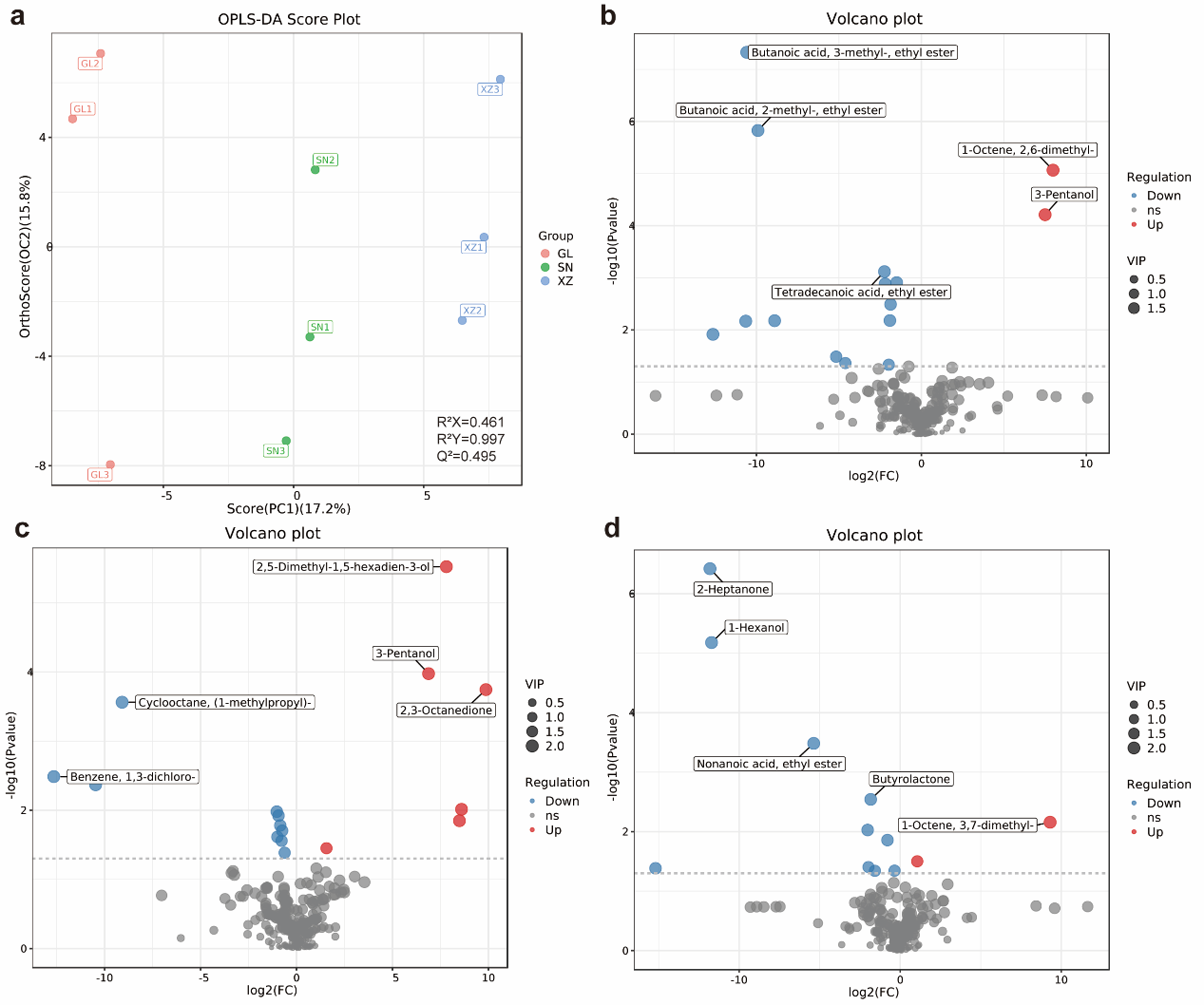


**Fig. S5.** OPLS-DA analysis of volatile organic compounds (VOCs). (a) OPLS-DA score plot of different groups. Volcano plots showing the differential VOCs levels between (b) GL and XZ, (c) SN and XZ, (d) GL and SN. The red nodes represent significantly up-regulated VOCs, blue nodes represent significantly down-regulated VOCs (p <0.05 and VIP >1), respectively. The grey nodes represent metabolites with no significance.

Table S1. The Relative Odor Activity Values (ROAVs) of key differential volatile organic compounds (VOCs).

| **Name** | **CAS** | **Formula** | **Range of Odor Min** | **Odor Character** | **GL_ROAV** | **SN_ROAV** | **XZ_ROAV** |
| --- | --- | --- | --- | --- | --- | --- | --- |
| 1-Heptanol | 111-70-6 | C7H16O | 3 | Grassy | 0.002203 | 0.004944 | 3.94E-05 |
| 1-Butanol, 3-methyl- | 123-51-3 | C5H12O | 1.69 | sweet, malty, rancid, rubber, | 0.004727 | 0.031657 | 0.021017 |
| Isopropyl Alcohol | 67-63-0 | C3H8O | 1000 | sharp, rubbing alcohol | 6.43E-06 | 1.09E-05 | 4.18E-05 |
| 1-Propanol, 2-methyl- | 78-83-1 | C4H10O | 10 | sweet, fusel, musty, alcohol, rubber, latex | 0.000188 | 0.000569 | 0.000164 |
| Ethanol | 64-17-5 | C2H6O | 24900 | Disolvent, Ethanol | 0.0006 | 0.001081 | 9.61E-05 |
| 2-Propanol, 2-methyl- | 75-65-0 | C4H10O | 3300 | sweet alcohol | 0.000144 | 0.000166 | 1.57E-05 |
| 1-Propanol | 71-23-8 | C3H8O | 53952.63 | Fruity, Floral, Grassy | 3.42E-08 | 7.58E-09 | 6.05E-08 |
| 1-Hexanol, 2-ethyl- | 104-76-7 | C8H18O | 0.198 | Rose, Green | 1.061722 | 1.381563 | 0.161104 |
| 2-Heptanol | 543-49-7 | C7H16O | 200 | Fruity, Mouldy, Musty, Mushroom | 1.9E-05 | 1.54E-05 | 3.44E-06 |
| 2,3-Butanediol | 513-85-9 | C4H10O2 | 4.5 | Fruits, Onion | 2.08E-06 | 0.230604 | 0.018636 |
| 1-Octanol | 111-87-5 | C8H18O | 0.9 | penetrating | 0.005389 | 0.034919 | 0.001397 |
| 1-Pentanol | 71-41-0 | C5H12O | 5.5 |  | 0.000943 | 0.003157 | 0.000259 |
| 1-Hexanol | 111-27-3 | C6H14O | 2.4 | green grass, plastic | 3.9E-06 | 0.021429 | 0.001506 |
| 1-Butanol | 71-36-3 | C4H10O | 3.3 | sweet, malty, alcohol, medicinal | 0.002401 | 0.002937 | 0.000417 |
| 2-Nonenal, (E)- | 18829-56-6 | C9H16O | 0.0002 | Fatty, Cucumber | 12.06143 | 36.33372 | 2.177626 |
| 2-Octenal, (E)- | 2548-87-0 | C8H14O | 0.003 | Nuts, Green, Fatty | 1.044907 | 3.33302 | 0.137374 |
| Propanal, 2-methyl- | 78-84-2 | C4H8O | 0.34 | pungent | 0.033714 | 0.259383 | 0.021713 |
| Octanal | 124-13-0 | C8H16O | 2.5 | Lemon, Citrus, Green Grass | 0.006915 | 0.024174 | 0.000753 |
| Hexanal | 66-25-1 | C6H12O | 20 | Green Grass, Fruity | 0.01119 | 0.016812 | 0.000965 |
| Benzeneacetaldehyde | 122-78-1 | C8H8O | 1 | Floral, Honey | 0.061015 | 0.227298 | 0.008178 |
| Methional | 3268-49-3 | C4H8OS | 7.12 | Cooked Potato | 0.0089 | 0.043643 | 0.000927 |
| Butanal, 2-methyl- | 96-17-3 | C5H10O | 0.001 | Cocoa, Almond | 13.72387 | 0.015254 | 2.050071 |
| Benzene, (1-methylethyl)- | 98-82-8 | C9H12 | 8 | sharp | 0.000269 | 0.000297 | 1.94E-05 |
| a-Methylstyrene | 98-83-9 | C9H10 | 20 |  | 0.000212 | 0.000222 | 4.14E-05 |
| Naphthalene | 91-20-3 | C10H8 | 1.9 | tar, creosote, mothballs, empyreumatic | 0.00076 | 0.000469 | 5.07E-05 |
| Benzeneacetic acid, ethyl ester | 101-97-3 | C10H12O2 | 406.83 | Floral, Rosy-Flavor, Honey | 1.38E-06 | 5.97E-06 | 9.13E-07 |
| Benzene, chloro- | 108-90-7 | C6H5Cl | 87 | almond-like, shoepolish | 0.0015 | 0.002012 | 0.000263 |
| Toluene | 108-88-3 | C7H8 | 21 | sour, burnt | 0.010208 | 0.022825 | 0.001356 |
| p-Cresol | 106-44-5 | C7H8O | 60 |  | 7.13E-05 | 0.000375 | 1.7E-05 |
| Phenol | 108-95-2 | C6H6O | 4.5 | medicinal, acid, ink, creosote, empyreumatic | 0.000964 | 0.002075 | 0.000205 |
| Benzene | 71-43-2 | C6H6 | 470 | aromatic, sweet, solvent, empyreumatic | 0.000169 | 0.000284 | 2.32E-05 |
| Benzene, 1,4-dichloro- | 106-46-7 | C6H4Cl2 | 121 | camphor, mothballs | 0.000112 | 0.000546 | 6.38E-06 |
| Butanoic acid, 3-methyl-, ethyl ester | 108-64-5 | C7H14O2 | 6.89 | Fruity, Apple-Flavor | 1.36E-06 | 0.001032 | 0.000313 |
| Propanoic acid, ethyl ester | 105-37-3 | C5H10O2 | 19019.33 | Ruity, Banana-Flavor | 3.49E-07 | 6.66E-07 | 1.49E-07 |
| Ethyl Acetate | 141-78-6 | C4H8O2 | 90 | fruity, sweet, fingernail polish, etherous | 0.000939 | 0.010418 | 0.001197 |
| Hexadecanoic acid, ethyl ester | 628-97-7 | C18H36O2 | 2 | Wax | 0.011669 | 0.064317 | 0.007341 |
| Tetradecanoic acid, ethyl ester | 124-06-1 | C16H32O2 | 500 | Sweet, Waxy | 9.08E-06 | 7.5E-05 | 6.92E-06 |
| Dodecanoic acid, ethyl ester | 106-33-2 | C14H28O2 | 500 | Sweet, Waxy, Floral | 1.79E-06 | 7.36E-06 | 8.36E-07 |
| Propanoic acid, 2-hydroxy-, ethyl ester | 97-64-3 | C5H10O3 | 128000 | Fruity, Slightly Fatty flavor | 2.35E-07 | 6.55E-06 | 3.31E-07 |
| Nonanoic acid, ethyl ester | 123-29-5 | C11H22O2 | 3150.61 | Floral, Fruity | 3.56E-09 | 3.48E-07 | 2.72E-08 |
| Ethyl formate | 109-94-4 | C3H6O2 | 2700 | aromatic | 1.09E-05 | 2.84E-06 | 9.65E-07 |
| Hexanoic acid, ethyl ester | 123-66-0 | C8H16O2 | 30 | Fruity, Green Apple | 0.000157 | 5.79E-05 | 1.33E-05 |
| Butanoic acid, 2-methyl-, ethyl ester | 7452-79-1 | C7H14O2 | 1.6 | Fruity | 5.84E-06 | 0.003187 | 0.000847 |
| Decanoic acid, ethyl ester | 110-38-3 | C12H24O2 | 420 | Brandy | 2.76E-06 | 7.82E-06 | 7.94E-07 |
| Butanoic acid, ethyl ester | 105-54-4 | C6H12O2 | 9.5 | Fruity | 0.001865 | 0.002503 | 0.000265 |
| Dimethyl sulfide | 75-18-3 | C2H6S | 0.12 | disagreeable, asparagus, putrid | 0.100425 | 0.224252 | 0.020568 |
| Ethanol, 2-butoxy- | 111-76-2 | C6H14O2 | 80 | sweet, ester, musty | 2.18E-05 | 9.68E-06 | 2.39E-06 |
| Propene | 115-07-1 | C3H6 | 10100 | gassy, aromatic | 5.36E-07 | 8.78E-07 | 1.14E-07 |
| Nonane | 111-84-2 | C9H20 | 2300 | gasoline | 5.37E-06 | 7.21E-06 | 6.95E-07 |
| Methyl vinyl ketone | 78-94-4 | C4H6O | 174 | pungent | 0.00018 | 0.000593 | 3.79E-05 |
| 5-Hepten-2-one, 6-methyl- | 110-93-0 | C8H14O | 50 | Herby, Green, Citrus, Musty, Lemongrass | 5.97E-05 | 0.000175 | 1.46E-05 |
| 1-Octen-3-one | 4312-99-6 | C8H14O | 0.005 | Mushroom-Like | 0.060614 | 0.915413 | 0.009573 |
| 2-Butanone | 78-93-3 | C4H8O | 70 | sweet, sharp | 0.04367 | 0.016042 | 0.000804 |
| Cyclohexanone | 108-94-1 | C6H10O | 52 | sweet, sharp | 1.69E-05 | 1.02E-06 | 2.32E-06 |
| Acetone | 67-64-1 | C3H6O | 400 | sweet, fruity, etherous | 0.000938 | 0.00098 | 0.000113 |
| Acetophenone | 98-86-2 | C8H8O | 0.24 | sweet, almond, pungent, oranges, river water | 0.110366 | 0.09263 | 0.017823 |
| 2-Hexanone, 5-methyl- | 110-12-3 | C7H14O | 2.1 | sweet, sharp | 0.006907 | 0.006893 | 0.00104 |
| 2-Pentanone | 107-87-9 | C5H10O | 28 | fingernail polish | 0.022115 | 0.030733 | 0.003815 |
| 2-Nonanone | 821-55-6 | C9H18O | 15 | Fruity, Floral, Fatty | 2.78E-05 | 0.000638 | 0.000144 |
| 2-Hexanone | 591-78-6 | C6H12O | 24 | sweet, paint | 0.001852 | 0.000229 | 0.000307 |
| 2-Heptanone | 110-43-0 | C7H14O | 0.75 | sweet, mushroom | 1.25E-05 | 0.06433 | 0.009268 |
| 2,3-Butanedione | 431-03-8 | C4H6O2 | 0.002 | pleasant, buttery | 100 | 66.67317 | 100 |
| p-Cymene | 99-87-6 | C10H14 | 11.4 |  | 1.75E-05 | 0.000213 | 2.24E-05 |
| 2-Propenal | 107-02-8 | C3H4O | 3.6 | pungent | 0.002929 | 0.031285 | 0.000218 |
| 3-Penten-2-one, 4-methyl- | 141-79-7 | C6H10O | 17 | sweet | 0.001623 | 0.001163 | 0.000157 |
| Trichloromethane | 67-66-3 | CHCl3 | 102 | sweet, etherous, suffocating | 4.81E-05 | 5.36E-05 | 5.97E-06 |
| Thiophene | 110-02-1 | C4H4S | 511 | Garlic, Alliaceous | 1.56E-06 | 5.22E-06 | 4.08E-07 |
| Thiophene, 3-methyl- | 616-44-4 | C5H6S | 360 | Plastic, Sulfurous | 1.23E-06 | 3.47E-06 | 2.46E-07 |
| Tetrahydrofuran | 109-99-9 | C4H8O | 92 | ether | 5.4E-05 | 0.000107 | 2.73E-06 |
| Thiazole | 288-47-1 | C3H3NS | 740 | Nut, Sulfur, Stink | 4.15E-06 | 1.67E-05 | 2.9E-07 |
| Butyrolactone | 96-48-0 | C4H6O2 | 35 | Wcreamy, Fatty | 0.000229 | 0.001782 | 0.000105 |
| Furan, 2-ethyl- | 3208-16-0 | C6H8O | 2.3 | Burnt, Sweet, Coffee-Like | 0.000533 | 0.001257 | 4.64E-05 |
| Pyrazine, methyl- | 109-08-0 | C5H6N2 | 121927.01 | Toasted Bread, Roasted Almonds, Fried Peanuts | 7.8E-08 | 8.87E-08 | 9.05E-09 |
| Octanoic acid, ethyl ester | 106-32-1 | C10H20O2 | 147 | Fruity | 1.05E-05 | 2.9E-05 | 1.78E-06 |
| Methane, isocyanato- | 624-83-9 | C2H3NO | 2140 |  | 5.91E-07 | 9.59E-07 | 6.79E-10 |
| Dimethyl trisulfide | 3658-80-8 | C2H6S3 | 0.36 | Sulfurous, Onion, Spicy | 0.006976 | 0.015289 | 0.00034 |
| Disulfide, dimethyl | 624-92-0 | C2H6S2 | 0.29 | garlic, putrid, asparagus | 0.018339 | 0.036214 | 0.000774 |
| Boron trifluoride | 7637-07-2 | BF3 | 1500 | pungent | 2.44E-06 | 4.06E-06 | 3.28E-07 |
| Phenylethyl Alcohol | 60-12-8 | C8H10O | 2600 | Floral | 3.6E-09 | 2.11E-06 | 7.9E-07 |
